# The influence of suture materials on the biomechanical behavior of suture-meniscal specimens: a comparative study in a porcine model

**DOI:** 10.1186/s43019-020-00053-4

**Published:** 2020-08-28

**Authors:** John Reza Matthews, Jiefei Wang, Jiwei Zhao, Melissa A. Kluczynski, Leslie J. Bisson

**Affiliations:** 1 Department of Orthopaedics, Jacobs School of Medicine and Biomedical Sciences, University at Buffalo, Buffalo, NY USA; 2 Department of Biostatistics, School of Public Health and Health Professions, University at Buffalo, Buffalo, NY USA

**Keywords:** Meniscal tears, Meniscus repair, Suture repair, Biomechanical analysis, Porcine

## Abstract

**Background:**

Repair of a meniscal tear is indicated in certain conditions. Despite extensive research on the biomechanics of various repair methods, there has been minimal investigation of whether the suture material influences the meniscal-suture construct. The purpose of this study was to compare the biomechanical properties of nine different suture materials under cyclic and load-to-failure conditions.

**Methods:**

Ninety porcine menisci were randomly allocated to simple suture placement using either Ultrabraid®, Ultratape®, Magnum Wire®, TigerWire®, TigerTape®, LabralTape®, Orthocord®, 0 FiberWire®, or 2-0 FiberWire®. Each suture-meniscus specimen underwent cyclic loading followed by load-to-failure testing. Elongation, maximum load to failure, stiffness, and mode of failure were recorded and compared between each suture type using non-parametric testing. Mean ± standard deviation was reported and the statistical significance was *p* < 0.05.

**Results:**

Elongation during cyclic loading was lowest with 2-0 FiberWire (0.95 ± 0.17 mm); this value was statistically significantly different than the results for all other sutures except 0 FiberWire® (1.09 ± 0.17 mm, *p* = 0.79), TigerWire® (1.09 ± 0.29 mm, *p* = 0.85), TigerTape® (1.39 ± 0.29 mm, *p* = 0.08), and LabralTape® (1.20 ± 0.33 mm, *p* = 0.41). The highest elongation was seen with Ultrabraid® (1.91 ± 0.34 mm); this value was statistically significantly greater than the results for all other suture materials except Orthocord® (1.59 mm ± 0.31 mm, *p* = 0.46) and Magnum Wire® (1.43 ± 0.25 mm, *p* = 0.14). Load to failure was highest for TigerTape® (287.43 ± 41.15 N), and this result was statistically significantly different than the results for all other sutures except LabralTape® (271.34 ± 48.48 N, *p* = 0.99) and TigerWire® (251.03 ± 25.8 N, *p* = 0.51). Stiffness was highest for LabralTape® (195.77 ± 49.06 N/mm), and this result was statistically significantly different than the results for all other sutures except TigerWire® (186.49 ± 19.83 N/mm, *p* = 0.45) and TigerTape® (173.35 ± 15.60 N/mm, *p* = 0.19). The majority of sutures failed by pullout (*n* = 46, 51%) or tearing (*n* = 40, 45%).

**Conclusion:**

Suture design and material affect the biomechanical behavior of porcine meniscal-suture specimens. LabralTape®, TigerWire®, and TigerTape® demonstrated better overall combinations of low elongation, high maximum load to failure, and high stiffness.

## Introduction

Meniscal tears are one of the most common pathologies encountered in the knee with an incidence of 66 per 100,000 [1]. Several studies have demonstrated an association between meniscal tears, osteonecrosis, high-grade chondral lesions, and progressive osteoarthritis [2–5]. These complications may be a result of increased tibiofemoral contact pressure secondary to decreased contact area, which has led to increased interest in meniscal repairs [6–10].

The goal of meniscal repair is an anatomically healed and biomechanically functional meniscus. The meniscus-suture interface is the most likely site of early failure. Numerous studies have compared different suture techniques (simple, locking loop, and other variations) to identify the strongest method to gain a stronghold in the meniscus [11–15]. However, only a few studies have investigated the effects of the suture material itself [16–19], despite the fact that suture design (cord versus tape) and material (polyester versus polyblend) have been shown to behave differently in tissues such as the rotator cuff [16, 20, 21].

The purpose of this study was to compare the biomechanical properties of nine different suture materials in a porcine meniscus model under both cyclic and load-to-failure conditions. The sutures used included Ultrabraid®, Ultratape®, Magnum Wire®, TigerWire®, TigerTape®, LabralTape®, Orthocord®, 0 FiberWire®, and 2-0 FiberWire®. We hypothesized that tape sutures would outperform wire sutures in ultimate load to failure and stiffness as a result of their thicker and broader design but with no significant differences with respect to elongation.

## Methods

### Porcine specimens

We obtained 130 fresh porcine menisci from Animal Biotech Industries, a local processing company. Porcine menisci were utilized because their mechanical properties are more consistent with those of young healthy adult human menisci compared to elderly cadavers, and porcine menisci are commonly used to evaluate meniscus repair in orthopedic research [19, 22–26]. Each meniscus was thoroughly inspected for tears or macroscopic signs of degeneration/abnormalities. Ninety menisci were chosen to compare the nine different suture materials, ten within each group, similar to prior studies evaluating biomechanical properties of suture materials [16, 19, 20, 26–28].

### Specimen preparation

Any soft tissue attachments including ligaments and connective tissue along the rim of the menisci were removed using a size 10 scalpel. Care was taken not to damage the menisci. All menisci were kept moist with saline-soaked gauze during the inspection, suturing, and testing period. All specimens were thawed once, as preparation and testing were performed during one laboratory session. Specimens were randomized into one of nine suture groups (Group 1: Ultrabraid®, Group 2: Magnum Wire®, Group 3: Ultra-tape®, Group 4: 2-0 FiberWire®, Group 5: 0 FiberWire®, Group 6: TigerWire®, Group 7: TigerTape®, Group 8: Orthocord®, Group 9: LabralTape®). The randomization process was designed to ensure that equal numbers of superior- and inferior-quality menisci were used in each suture group through random selection from a bag that contained all the specimens. We did this to account for any intra-meniscal variability that could introduce selection bias. All specimens were analyzed, prepared, and tested by the same investigator. This testing protocol has been used in other biomechanical suture studies [20].

**Fig. 1:**
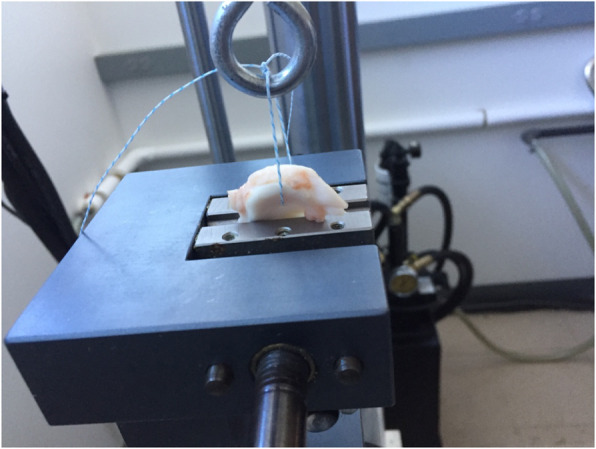
MTS device with porcine meniscus in place. Depicts the meniscal suture specimen within the MTS device while testing

The experiment was designed to specifically investigate the biomechanical properties of each suture material. In order to eliminate the effect of passing a different sized needle through each meniscus, a free tapered needle was used in the passing of all suture material. To eliminate the variability of more complex suture techniques and remain consistent with prior studies, a simple stitch was used [16]. Using a micro caliper, each suture was passed 1 cm away from the root at a point 1 cm anterior from the posterior edge and tied with five square knots, using two half hitches, a locking half hitch, and then two additional alternating locking half hitches. The suture loop was then placed over an S-hook and the menisci placed into the clamp 1 cm from the suture-meniscus interface. The experimental construct is demonstrated in Fig. 1. This technique has been used in prior studies [20, 21].

### Testing

**Fig. 2:**
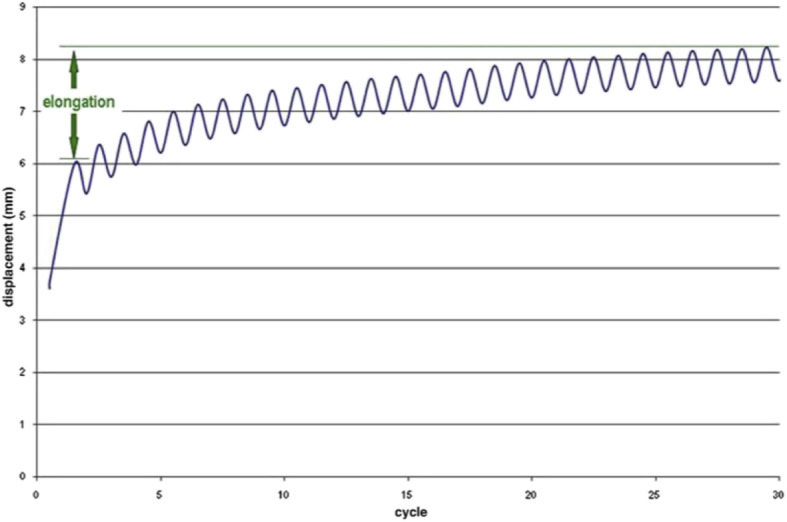
Assessment of elongation; typical cycle-versus-elongation graph [20]

All testing was performed at room temperature with the suture loop placed on the S-hook attached to the load cell of a Bionix MTS (measure, test, simulate) machine (MTS Systems, Eden Prairie, MN, USA). The testing protocol was adopted from prior studies evaluating meniscal repair techniques thought to simulate in vivo loads during the early postoperative period [16, 18, 19, 22, 24]. Prior to cyclic loading, a preload of 5 N was placed for 30 s, followed by 30 loading cycles ranging between 5 and 30 N at 0.25 Hz, by use of a half-sinusoidal waveform similar to the procedure in previous studies [20]. The amount of 30 cycles was chosen based on prior studies demonstrating stabilization of the displacement-versus-time curve between 20 and 30 cycles as represented in Fig. 2 [20]. This cyclic protocol has been used by other investigators [21, 29, 30]. Upon completion of cyclic loading, each specimen underwent load to failure at 5 mm/s. The maximum load to failure was considered as the peak force recorded. The mode of failure was determined by visual inspection and included suture breakage, suture pullout from the meniscus, and knot failure.

### Data collection and statistical analysis

The number of cycles and the displacement were recorded simultaneously by use of data acquisition software. Elongation was calculated as the difference between the displacement at the end of the 5-N preload and the maximum displacement during the 30th cycle [20]. The stiffness (in newtons/millimeter) of each suture was calculated by determining the slope of the best-fit line on the load-versus-displacement curve.

For each suture type, we analyzed three continuous variables including the mean ± standard deviation of elongation (millimeters), ultimate tensile load (newtons), and stiffness (newtons/millimeter). All statistical analyses were performed with SAS version 9.4 (SAS Institute, Cary NC, USA), and statistical significance was considered as *p* < 0.05. Wilcoxon analysis and Kruskal-Wallis tests were performed to determine the presence of an overall difference within each variable (elongation, load to failure, and stiffness). To determine if there were statistically significant differences between suture groups, a non-parametric pairwise comparison test using the Dwass, Steel, Critchlow-Fligner (DSCF) method was performed. The null hypothesis was rejected if the *p* value was < 0.05, which would indicate that there were statistically significant differences within the nine groups. In order to make a true comparison of suture behavior without contamination by knot failures, the data analysis was performed a second time excluding all knot failures.

## Results

### Elongation by suture type

**Fig. 3:**
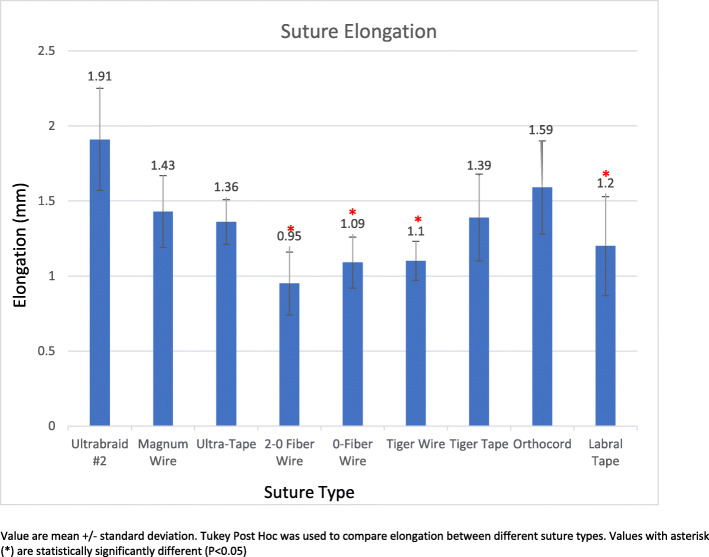
Suture elongation. Depicts the suture elongation (in millimeters) per suture group

Figure 3 demonstrates the mean elongation (in millimeters) by suture type. Ultrabraid #2 demonstrated the greatest elongation at 1.91 mm ± 0.34 mm, which was statistically significantly greater compared to the values for all other suture materials (*p* < 0.05) except Orthocord (1.59 mm ± 0.31 mm, *p* = 0.46) and Magnum Wire (1.43 mm ± 0.25 mm, *p* = 0.14). The lowest elongation was seen in 2-0 FiberWire at 0.95 ± 0.17 mm which was not statistically significantly different from the values for 0 FiberWire (1.09 ± 0.17 mm, *p* = 0.79), TigerWire (1.09 ± 0.29 mm, *p* = 0.85), TigerTape (1.39 mm ± 0.29 mm, *p* = 0.08), and LabralTape (1.20 ± 0.33 mm, *p* = 0.41).

With the exclusion of four knot failures, the only statistically significant difference in elongation was seen with Orthocord (1.59 mm ± 0.31 mm), which demonstrated greater elongation in comparison with LabralTape (1.20 ± 0.33 mm, *p* = 0.02).

### Maximum load to failure by suture type

**Fig. 4:**
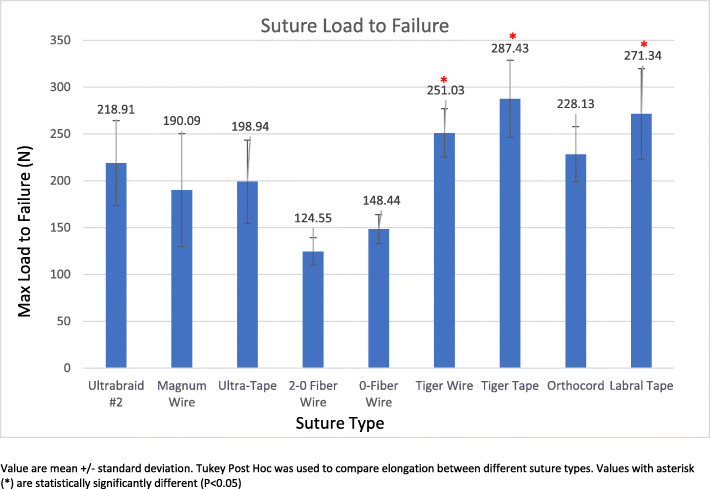
Suture load to failure. Depicts the load at which the suture failed (in newtons) per suture group

Figure 4 represents the mean maximum load to failure (in newtons) and the standard deviation for each suture type. TigerTape demonstrated the highest load to failure at 287.43 ± 41.15 N, which was a statistically significantly greater value than those for all suture materials (*p* values < 0.05) except TigerWire (251.03 ± 25.8 N, *p* = 0.51) and LabralTape (271.34 ± 48.48 N, *p* = 0.99). 2-0 FiberWire demonstrated the lowest maximum load to failure at 124.55 ± 14.69 N; this value was statistically significantly different from those of all other suture materials (*p* values < 0.05) except Magnum Wire (190.09 ± 61.44 N, *p* = 0.07) and 0 FiberWire (148.44 ± 15.41 N, *p* = 0.49).

With the exclusion of four knot failures, there were two statistically significant differences in ultimate tensile load. The first was seen between TigerTape (287.43 ± 41.15 N) and Ultrabraid #2 (218.91 ± 45.68 N, *p* = 0.049). Additionally, Ultratape (198.94 ± 39.23 N) and TigerWire (251.03 ± 25.87 N, *p* = 0.049) demonstrated a statistically significant difference.

### Stiffness

**Fig. 5:**
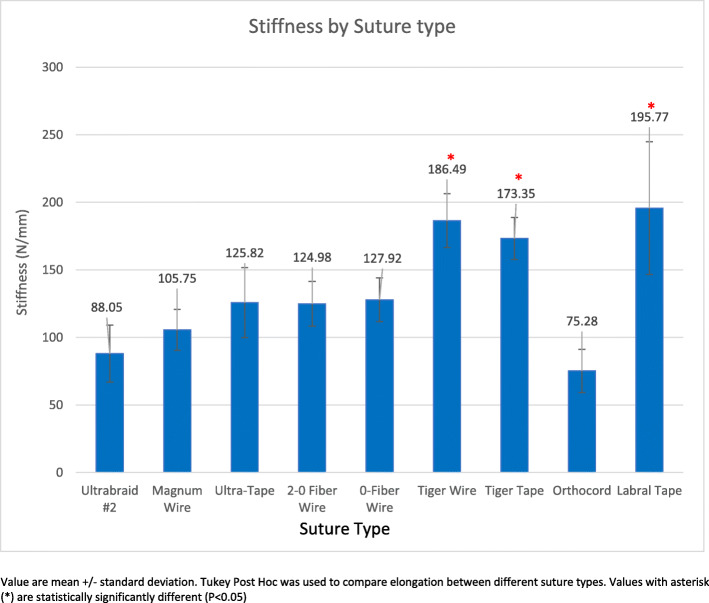
Suture stiffness. Depicts the stiffness in newtons per millimeter per suture group

Figure 5 demonstrates the mean stiffness (in newtons/millimeter) for each suture type. TigerTape (173.35 ± 15.60 N/mm) and TigerWire (186.49 ± 19.83 N/mm) demonstrated statistically significant differences from all other suture materials (*p* < 0.05). LabralTape (195.77 ± 49.06 N/mm) initially demonstrated no statistically significant differences from the eight other sutures until the analysis was repeated with the exclusion of knot failures. With the exclusion of four knot failures, LabralTape demonstrated statistically significant differences with all other sutures (*p* < 0.05) except TigerWire (*p* = 0.45) and TigerTape (*p* = 0.19). Orthocord demonstrated the lowest stiffness at 75.28 ± 16.01 N/mm; this value was statistically significantly lower than those for all other suture materials (*p* < 0.05) except Ultrabraid #2 (88.05 ± 21.03 N/mm, *p* = 0.97).

With the exclusion of four knot failures, in addition to the changes seen with LabralTape, there were two changes seen with Ultrabraid #2. First, Ultrabraid #2 demonstrated a statistically significant difference in stiffness with Magnum Wire (105.75 ± 12.68 N/mm, *p* = 0.03) but no longer a statistically significant difference with 0 FiberWire (127.92 ± 16.23 N/mm, *p* = 0.06). The remaining sutures did not show any statistically significant changes.

### Mode of failure

Table 1 demonstrates the mode of failure and the characteristics for each suture material. The majority of sutures failed by suture pullout (51%) or suture breakage (45%), with only four knot failures (4%). The only statistically significant difference between mode of failure was evident in the two sutures with zero failures due to breakage (Ultratape and LabralTape) compared to 2-0 and 0 FiberWire with 10 and 9 failures secondary to suture breakage, respectively.

**Table 1:** Method of failure and suture characteristics

Suture	Method of failure				Suture characteristics	
	Suture pullout	Suture breakage	Knot failure	Load at knot failure (N)	Bioabsorbability	Material
**Ultrabraid**	6 (60%)	3 (30%)	1 (10%)	162.17	Non-absorbable	Braided UHMWPE
**Magnum wire**	6 (60%)	3 (30%)	1 (10%)	142.1	Non-absorbable	Braided polyethylene
**Ultratape**	9 (90%)	0 (0%)	1 (10%)	168.89	Non-absorbable	Smooth UHMWPE
**2- 0 FiberWire**	0 (0%)	10 (100%)	0 (0%)	None	Non-absorbable	Braided polyester with long-chain polyethylene core
**0 FiberWire**	1 (10%)	9 (90%)	0 (0%)	None	Non-absorbable	Braided polyester with long-chain polyethylene core
**TigerWire**	4 (40%)	6 (60%)	0 (0%)	None	Non-absorbable	UHMWPE with long-chain polyethylene core and additional black marker strand
**TigerTape**	6 (60%)	4 (40%)	0 (0%)	None	Non-absorbable	Broad UHMWPE with long-chain polyethylene core and additional black marker strand
**Orthocord**	5 (50%)	5 (50%)	0 (0%)	None	Partially absorbable	Braided UHMWPE (45%) with PDS core (55%)
**LabralTape**	9 (90%)	0 (0%)	1 (10%)	181.0	Non-absorbable	Smooth UHMWPE

*UHMWPE* ultra-high molecular weight polyethylene, *PDS* polydioxanone

With the exclusion of knot failures, there were no statistically significant differences in mode of failure.

## Discussion

In this study, we evaluated the biomechanical properties of nine different sutures using a porcine meniscus model. At the completion of this study, the most important findings were no significant differences in elongation between wire and tape sutures; however, tape sutures did demonstrate a trend towards greater maximum load to failure and stiffness, supporting our hypothesis.

Overall, LabralTape, TigerWire, and TigerTape demonstrated a better combination of low elongation, high ultimate load to failure, and high stiffness. Despite both 2-0 and 0 FiberWire demonstrating low elongation, their ultimate tensile strength was significantly lower than those of the other suture materials, resulting in the majority failing due to breakage, as seen in Table 1. The low elongation seen with the FiberWire sutures is likely a result of the braided polyester jacket. However, the FiberWire maximum load to failure was inferior to those of the remaining sutures containing ultra-high molecular weight polyethylene (UHMWPE) (Table 1). The sutures containing UHMWPE, commercially referred to as Dyneema, are manufactured through a gel-spinning process and are capable of absorbing large amounts of energy while remaining flexible [31]. Therefore, the sutures containing UHMWPE in their core and jacket demonstrated very high tensile strength (Table 1). Ultrabraid, despite being a UHMWPE suture, does not contain a UHMWPE central core when viewed in transverse cross section, which likely resulted in slightly inferior maximum load to failure [31]. Futhermore, despite Orthocord containing a braided UHMWPE jacket, its core consists of a monofilament resulting in a maximum load to failure in between that of the FiberWire and tape sutures containing 100% UHMWPE. The Orthocord configuration with a PDS (polydioxanone) core was designed to leave a lower profile once the core suture dissolves but retain its strength with the outer UHMWPE sleeve and have less bacterial adherence [32]. TigerWire and TigerTape sutures, when compared to FiberWire, contain the same polyethylene core but have a UHMWPE jacket and an additional black strand that likely contributed to their higher tensile strength but similar elongation. When comparing the mode of failure, sutures containing mainly polyester (2-0 and 0 FiberWire) primarily failed by breakage due to their lower ultimate load of failure, compared to sutures containing UHMWPE in their jacket and core (LabralTape/Ultratape), which failed primarily by pullout. Sutures containing a combination of both (TigerWire, TigerTape, Orthocord) failed by both mechanisms. As the majority of these new sutures are non-bioabsorbable, it is critical that we understand the properties and characteristics of the implanted material.

There have been very few prior biomechanical studies evaluating characteristics of suture materials. The ideal suture for meniscal repairs should have a high load to failure to prevent detachment during the healing process, low displacement to prevent suture elongation resulting in non-anatomic healing, and high stiffness to avoid deformation under loading conditions [33]. Post et al. evaluated both repair technique (mulberry knot, horizontal and vertical mattress) and three different suture materials (2-0 Ethibond, 0 PDS, and 1 PDS) for meniscal repairs in a porcine model [25]. They concluded that 1 PDS had the greatest load to failure using the vertical mattress technique [25]. Feucht et al. compared the biomechanical properties of PDS®, Ethibond®, FiberWire®, and FiberTape® and found FiberTape to be the strongest and stiffest material with failures occurring at the suture-meniscus interface, as compared to PDS and Ethibond, which failed secondary to suture breakage [16]. Our study demonstrated similar findings with the highest load to failure in tape sutures and failures occurring due to suture pullout while no tape sutures failed by breakage. Similar to the results of Feucht et al., elongation was lowest in the FiberWire sutures, although our study included a greater variety of suture materials.

A previous porcine meniscus study demonstrated that elongation and extrusion in excess of 3 mm has significant effects on meniscal function and is linked to increased articular cartilage loss and osteophyte formation [34, 35]. None of the sutures in our study demonstrated an elongation of 3 mm; however, most tape sutures demonstrated greater elongation than that of the cord sutures except LabralTape. We believe this is due to their thicker composite resulting in increased slack and decreased friction within the knot. In order for a knot to be secure, it relies on both knot security, defined as effectiveness of resisting slippage when loaded, and loop security, which is the ability to maintain a tight suture loop as a knot is tied [35–37]. The thicker nature of the tape sutures may result in greater slack between ties and thus greater elongation during cyclic loading and higher tendency for knot failure. There were no knot failures with the three wire sutures (2-0 and 0 FiberWire, TigerWire). However, the thicker composite of the tape sutures was advantageous during maximum loading. With higher maximum load to failure, the weak point for tape sutures was mainly at the suture-meniscus interface with suture pullout being the primary method of failure. This higher load-to-failure strength may provide protective benefits during weight bearing and potentially allow for earlier range of motion and return to activity. With a recent shift from braided polyester sutures and monofilaments to high-strength polyblend sutures [38], determining the best suture may provide additional benefits for an optimal outcome.

To date, there are no recommendations regarding the choice of suture material for repair of meniscus tears. In our study, LabralTape, TigerTape, and TigerWire demonstrated better overall combinations of low elongation, highest loads to failure, and stiffness. In the clinical setting however, it is unknown whether younger patients with more mobile tissue are more suitable for a stiffer material to counterbalance the more mobile tissue, whereas older more frail tissue may benefit from a less stiff suture construct to limit suture pullout.

This study has several limitations. First, we used porcine menisci rather than human specimens because cadaveric menisci tend to be older with expected degenerative changes. Porcine menisci have also been used in previous studies evaluating the biomechanics of various suture materials, which allows easier comparison [16, 19, 22, 26, 39]. Secondly, we did not investigate the physiologic effects of the suture material, which may be advantageous or disadvantageous in the clinical setting. The loading pattern in our study may not reflect in vivo repairs, which may be subject to more complicated loading patterns. However, our testing method was similar to those of other studies [20, 21, 24, 25, 40]. Our model used a simple stitch in order to minimize the influence of more complex techniques since the primary focus of this study was to evaluate the biomechanical properties of different suture materials. The arthroscopic instruments used in the clinical setting were not used in the lab because we believed the methods used would magnify the effect of biomechanical differences between suture materials. Therefore, the results of this study may not entirely be extrapolated to other suture techniques. This study also did not analyze the specific characteristics of each suture material. Further investigation of in vivo differences among suture materials is warranted.

## Conclusion

In order to avoid the consequences related to meniscal deficiencies, surgeons may opt to repair meniscal tears. For optimal meniscal healing, it is essential to utilize a suture-meniscus construct with low elongation to prevent gap formation and with a high load to failure. TigerWire®, TigerTape®, and LabralTape® demonstrated better overall biomechanical characteristics with the lowest elongation during cyclic loading, highest maximum load to failure, and highest stiffness. These three sutures may be the preferred suture material for meniscal repairs.

## Data Availability

The data are available; materials are disposed of after testing.

## Abbreviations

PDS: Polydioxanone
UHMWPE: Ultra-high molecular weight polyethylene
